# Barriers and Facilitators to Sustaining School-Based Mental Health and Wellbeing Interventions: A Systematic Review

**DOI:** 10.3390/ijerph19063587

**Published:** 2022-03-17

**Authors:** Anna Moore, Emily Stapley, Daniel Hayes, Rosa Town, Jessica Deighton

**Affiliations:** 1 Evidence Based Practice Unit (EBPU), University College London, London N1 9JH, UK; emily.stapley@annafreud.org (E.S.); daniel.hayes@annafreud.org (D.H.); rosa.town.13@ucl.ac.uk (R.T.); jessica.deighton@annafreud.org (J.D.); 2 Anna Freud National Centre for Children and Families (AFNCCF), London N1 9JH, UK; 3 Research Department of Clinical, Educational and Health Psychology, Faculty of Brain Sciences, University College London, London WC1E 6BT, UK

**Keywords:** sustainability, school-based, mental health, wellbeing, intervention

## Abstract

Despite an increasing focus on schools to deliver support and education around mental health and wellbeing, interventions are often not sustained beyond initial funding and research. In this review, the barriers and facilitators to sustaining mental health and wellbeing interventions in schools are explored. A systematic review was conducted using keywords based on the terms: ‘sustainability’, ‘school’, ‘intervention’, ‘mental health’, and ’emotional wellbeing’. Six online databases (PsycINFO, Embase, MEDLINE, British Education Index, ERIC, and Web of Science) and relevant websites were searched resulting in 6160 unique references. After screening, 10 articles were included in the review and extracted data were qualitatively synthesized using thematic analysis. Data synthesis led to the identification of four sustainability factors at the school level (school leadership, staff engagement, intervention characteristics, and resources) and one at the wider system level (external support). These factors were separated into 15 themes and discussed as barriers and facilitators to sustainability (for example, school culture and staff turnover). Most articles included no definition of sustainability, and nearly all barriers and facilitators were discussed at the school level. The findings suggest that more longitudinal and theory-driven research is required to develop a clearer picture of the sustainability process.

## 1. Introduction

Improving young people’s mental health and wellbeing has been identified as one of the key public health issues of our time, and recent prevalence findings show that one in eight young people experience mental health problems [1]. In the United Kingdom (U.K.), research has found that emotional problems such as anxiety and depression are the most common issues experienced by young people, followed by behavioural problems [2]. It is widely acknowledged that these difficulties may have a costly negative impact on educational attainment, drug use, criminality, physical health, poorer employment outcomes, not in education, employment or training (NEET) status, and financial difficulties [3,4,5,6]. The World Health Organisation (WHO) has called for a coordinated response from different sectors of society, noting that “among all the sectors that play critical roles in adolescent health, education is key” [7] (p. 8). 

Internationally, schools are increasingly being perceived as important sites to embed mental health and wellbeing prevention programmes, and a number of recent reviews have highlighted areas of promise in relation to school-based mental health support [7,8,9]. A recent systematic review carried out by the Early Intervention Foundation identified programmes for social and emotional skills and cognitive behavioural therapy (CBT) interventions for internalising symptoms to be particularly effective in improving mental health outcomes for children and young people [9]. Another review focusing on effective universal interventions for mental health and wellbeing noted that while the evidence base was still limited, there were a number of common practices, namely, that interventions were school-based and skills-based, often drew on CBT principles, included a discrete number of sessions, and were designed to be fun and engaging [10]. 

This emerging evidence base has coincided in the U.K. with an increased policy focus on schools as a site for mental health support. Recent examples in England include the Transforming Children and Young People’s Mental Health Green Paper, which provided a framework for school mental health support through senior mental health leads in schools and mental health support teams, and the new Relationships, Sex and Health Education guidance, which incorporated the teaching of mental health into the curriculum guidelines [11,12]. There have also been several government-commissioned programmes that attempt to embed mental health interventions in schools. Examples include Social and Emotional Aspects of Learning (SEAL), Targeted Mental Health in Schools (TaMHS) and, more recently, large-scale research programmes to test the effectiveness of universal mental health programmes [13,14,15,16]. While popular during their initial delivery, many of these programmes report significant variation in intervention fidelity and seem to have a relatively short shelf-life once initial funding finishes [13]. 

Lack of sustained delivery is common across contexts and countries; whilst governments across the globe invest substantially in the roll-out of mental health promotion programmes in schools, there are “concerning reports, nationally and internationally, about poor programme sustainability once start-up enthusiasm and resources are exhausted” [17] (p. 2). It is clear from the considerable investment of resources and the evidence of the success of these interventions that it is in the best interest of the health and education systems, as well as individual schools and pupils, to achieve long-term sustainability of such programmes. Although this issue in schools is well recognised, and there is wider research literature on what affects sustainability and how sustainability of effective programmes can be improved, there has been limited focus on educational settings [18,19].

Historically, research on sustainability has been fragmented and drawn from a variety of settings (predominantly in healthcare) which may or may not have parallels with the school context [18]. For wider health programmes, common factors that promote sustainability include workforce capacity, programme champions, organisational culture and context, evaluation and feedback, intervention effectiveness, staff turnover, and the wider political climate [18,20]. In a recent review focusing specifically on health interventions in school settings, Herlitz et al. [19] found many similar factors affecting sustainability. However, this review also noted that academic education was, at times, prioritised over health interventions and that staff sometimes lacked confidence delivering health promotion programmes that were outside of their usual expertise [19]. 

There is an argument therefore to suggest that topics that are traditionally considered outside the scope of schools may be more difficult to sustain in these contexts. As previously mentioned, schools’ mental health remit has recently changed to include educating pupils about mental health and wellbeing and providing support to children and young people. Consequently, it is important to understand just how much these wider factors around the sustainability of health interventions are relevant to mental health interventions in the school context. This review aims to contribute to the literature by identifying studies carried out in this area and addressing the question: what are the barriers and facilitators to sustaining school-based mental health and emotional wellbeing interventions?

## 2. Materials and Methods

A protocol for this systematic review was published on PROSPERO in August 2020 (ref: CRD42020189253), and relevant PRISMA guidelines for reporting were followed [21].

### 2.1. Definitions

Two of the key constructs in this review, ‘mental health and emotional wellbeing’ and ‘sustainability’, are not consistently defined and used in the literature [18,22,23,24]. The term ‘wellbeing’ is interpreted differently in different fields and research disciplines, and the relationship between mental health and wellbeing is poorly defined [22]. However, for this systematic review, the constructs are used together in an attempt to capture articles on the range of interventions (discussed above) that are currently taking place in schools. As a result, specific terms associated with internalising (e.g., depression, anxiety, eating disorders) and externalising (e.g., behaviour problems, aggression, substance abuse) problems, along with broader terms such as ‘mental health’ and ‘wellbeing’, were included. 

Of the various definitions and frameworks used to conceptualise the term ‘sustainability’, Wiltsey-Stirman et al. [18] identified the most cited definition in the literature as that proposed by Scheirer [25]. This defines sustainability on three different levels: (a) individual level: continuing to deliver the desired outcomes or benefits for individual community members; (b) organisational level: an organisation maintaining the programme or intervention in an identifiable form, even if modified; and (c) community level: maintaining the capacity of a community/region/nation to deliver programme activities after an initial implementation period is over [25]. This review draws on this definition but does not focus on the individual outcomes at the pupil level (a), as these outcomes are sometimes included in long-term follow-ups of intervention effectiveness studies. Instead, this review focuses on addressing the gap in understanding around sustained delivery of school-based mental health and emotional wellbeing interventions, and consequently, it centres on the organisational (b) and community (c) level factors that may affect sustainability. 

This review employs the WHO’s definition of a health intervention as “an act performed for, with or on behalf of a person or population whose purpose is to assess, improve, maintain, promote or modify health, functioning or health conditions” [26].

### 2.2. Study Eligibility

Studies were considered for inclusion if they met the following criteria:

(i) The study focused on the sustainment of a school-based mental health or emotional wellbeing intervention, and research was carried out after the end of the initial implementation period (when initial funding and/or external support had ended).
(ii) The intervention:
  • Targeted school-aged children and young people (CYP; between 4 to 18 years of age);
  • Aimed to improve mental health and emotional wellbeing outcomes;
  • Was delivered during school hours primarily by staff in or associated with the school (e.g., teachers, pastoral, managerial or administrative staff, health or wellbeing professionals employed or commissioned to work with the school) or students (e.g., peer mentors).
(iii) Participants in the research were involved as receivers, developers, or evaluators (e.g., intervention developers, school staff, or researchers) of the school-based mental health or emotional wellbeing intervention.
(iv) The study used quantitative or qualitative empirical methods to explore sustainability, or was a systematic review synthesising empirical studies.
(v) The study was published in English since the year 2000

Studies were excluded if they reported only on the initial implementation phase of delivery or reported intentions to sustain or continue activities with no research conducted after initial funding and external support had ended. Studies that only reported outcomes on the individual level (pupil) at long-term follow-up, with no mention of programme activities, were also excluded. Interventions that were delivered primarily outside of school hours or by external providers (e.g., an after-school club in a community centre) were excluded as this review focused specifically on programmes delivered in the context of mainstream education. 

### 2.3. Search Strategy

The following electronic databases were searched on 3rd and 5th March 2021 for potentially relevant studies: PsycINFO, MEDLINE, Embase, British Education Index, ERIC, and Social Sciences Citation Index and Conference Proceedings Citation Index – Social Science and Humanities (Web of Science). Each database was searched to find articles containing terms related to four key components: sustainability, school, intervention, and mental health/emotional wellbeing (see Supplementary Material File S1 for an example search strategy). A grey literature search was also conducted by identifying key websites (see Supplementary Material File S1). Additionally, the reference sections of included studies were checked, and a citation search was conducted on Google Scholar.

### 2.4. Screening

Results from the database searches were uploaded to the review management software EPPI-Reviewer Web [27] and duplicates were removed. All titles and abstracts were screened by the lead author (AM). The fourth author (RT) independently screened 10% of the studies at the title and abstract stage, and an interrater reliability analysis using the kappa statistic was performed to determine consistency among raters (k = 0.84, *p* = 0.001). Any discrepancies were resolved through discussion with the other authors, and AM went on to screen the remaining titles and abstracts. Full-text copies of the remaining articles were retrieved and screened by AM. RT also assessed 10% of the full texts (k = 0.82, *p* = 0.001), and the final decisions on included articles were made collectively as a research team.

### 2.5. Quality Assessment

Quality assessment of included articles was conducted using the Mixed Methods Appraisal Tool (MMAT; [28]). This tool, designed to appraise the methodological quality of research studies, allows for simultaneous evaluation of all empirical literature (i.e., qualitative, quantitative, and mixed methods studies) which was appropriate for this review. The MMAT has high intraclass correlation and has been shown to be efficient and user-friendly [29]. Quality scores for each article ranged from meeting none of five criteria (zero) to meeting all five criteria (five). Articles scoring zero to one are described as ‘low’ quality, two to four as ‘medium’ quality and five as ‘high’ quality.

### 2.6. Data Extraction and Data Synthesis

A data extraction table was designed specifically for this review, drawing on best practice guidance [30]. Extracted variables included: geographical location; description of school-based intervention (aim, population, and design); sustainability definition; sustainability study population, sample size, and data collection methods; and factors affecting sustainability. 

Due to the lack of homogenous quantitative studies, it was not possible to conduct a meta-analysis; no two studies in this review used similar measures that could be compared quantitatively. Consequently, the results sections of quantitative, qualitative, and mixed-methods studies were imported into the data analysis software NVivo [31]. A thematic analysis was conducted by the primary researcher (AM) using the six steps identified by Braun and Clarke [32]. AM read and re-read the data (Step 1) and conducted line-by-line inductive coding of the included results sections (Step 2). ES also conducted the same coding process on 20% of the articles, and potential themes were created and discussed (Step 3). AM then continued reviewing and refining themes and created a thematic map and detailed corresponding table. The synthesis was then discussed with ES, JD, and DH before the final write up of the results section (Step 6).

## 3. Results

**Figure 1:**
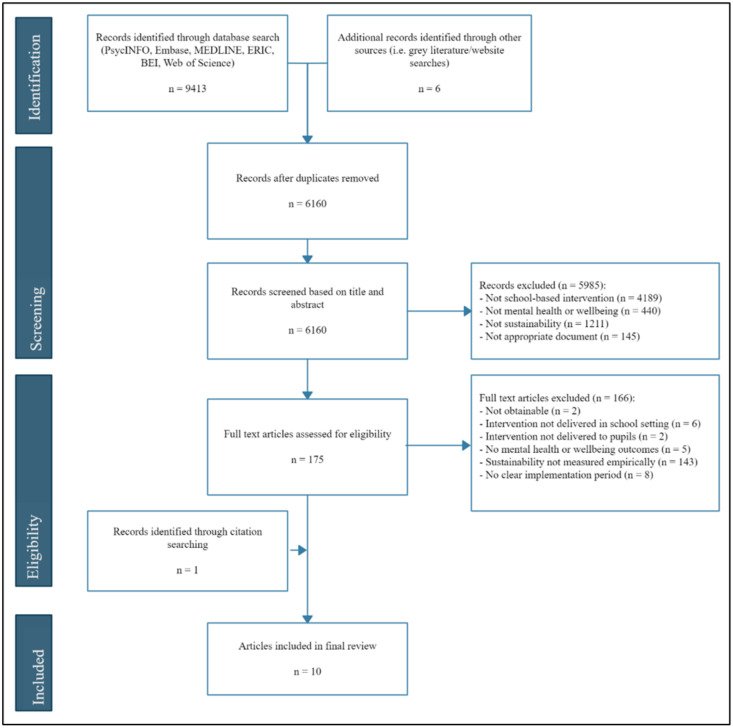
PRISMA flow diagram of the study selection process.

Of the 6160 articles identified through database searching, 10 articles met inclusion criteria and provided information that could be extracted on factors affecting sustainability (see Figure 1).

### 3.1. Study Characteristics

The country with the majority of the included studies was the United States (U.S.), with six articles [33,34,35,36,37,38]. The remaining studies were conducted in Germany [39], Norway [40], the Netherlands [41] and the U.K. [42].

Studies presented findings on a range of different school-based programmes, including interventions aimed at reducing symptoms of eating disorders and weight control behaviours [34,39]; CBT-informed interventions to treat pupils experiencing trauma, anxiety, and depression [33,37,38]; interventions addressing behaviour problems [35,36,40,41]; and a broader screening tool focused on social, emotional, and mental health needs [42]. See Table 1 for a summary of intervention characteristics and reported effectiveness.

### 3.2. Sustainability Terms and Definitions

Four articles used alternative terms to refer to sustainability, including ‘long-term implementation’ [39], ‘continuation’ [40], ‘maintenance’ [35], and ‘de-adoption’ [37]. LoCurto et al. [38] referred to ‘sustained use’ of the intervention, while the remaining five articles used the term ‘sustainability’ throughout [33,34,36,41,42]. 

The timeframe between the initial implementation period and the sustainability evaluation varied between studies (see Table 2). Two studies evaluated sustainability of the programme less than a year after initial delivery [35,42], four took place one to two years later [33,34,37,41], and four studies were conducted three to ten years after the initial implementation period [36,38,39,40]. 

Five studies provided no definition of sustainability but referred only to activities being ‘sustained’ or ‘maintained’ at follow-up [34,35,38,39,42]. While several studies discussed prevailing implementation and sustainability theories or frameworks in their introductions, Dijkman et al. [41] and Loman et al. [36] were the only papers to develop a clear theoretical framework which was then used to guide research processes and analysis. For an overview of the different terms and frameworks used, see Table 2. 

### 3.3. Study Design

Five studies were conducted using qualitative methods [33,37,39,40,42] and two of the included studies used solely quantitative data collection methods [36,38]. Qualitative studies consisted of interviews with school staff, including teachers, headteachers, school clinicians, and psychologists. The remaining three studies used mixed methods, combining a checklist or questionnaire with qualitative interviews [34,35,41]. Table 3 outlines the study design and participants.

### 3.4. Quality Assessment

The quality of three of the qualitative studies was high, with rigorous data collection methods and coherent analysis and interpretation [33,37,39]. The quality of the remaining studies was lower. For the qualitative studies, this was mainly due to a lack of clarity in reporting of methods. Quantitative studies had issues with nonresponse bias, while mixed methods studies did not adequately integrate the qualitative and quantitative components of their design (see Table 3 for further details). 

### 3.5. Synthesis of Barriers and Facilitators to Sustainability

**Figure 2:**
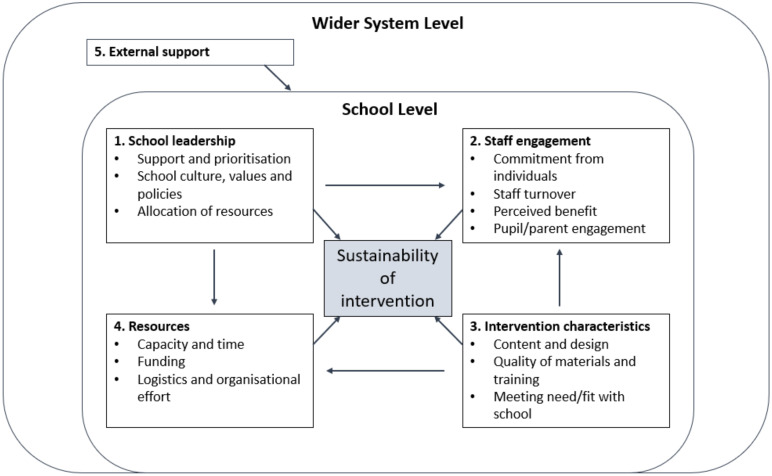
Thematic map of factors affecting sustainability.

For a list of the factors affecting sustainability that were discussed in each article see Table 4. The overarching factors, themes, and subthemes are described in detail below and the links between themes are portrayed visually in Figure 2. 

Figure 2 is a visual representation of the various themes and subthemes outlined in Table 4. The arrows between the key sustainability factors identified here highlight the directional relationships between themes. For example, the theme around support and prioritisation of the intervention by school leaders (1) was linked to the allocation of resources (4), affecting the capacity of staff. Similarly, school culture, which was shaped by the school leadership (1), fed into staff engagement (2). The characteristics of a given intervention (3) affected the logistics and organisational effort (4) required for successful delivery, along with the engagement by individual staff members (2). Each theme is discussed in detail below, starting with the findings at the school level and then moving onto higher-level factors.

### 3.6. School Leadership

The influence of the school leadership team on the sustainability of an intervention was cited as a key factor in nearly all the papers included in this review. This factor is broken down into three themes below, with subthemes italicised in the text.

#### 3.6.1. Support and Prioritisation

School leaders *prioritising the intervention* was identified as a key facilitator of sustainability, with teachers stating that leadership support was crucial to ensure that intervention activities would continue in the school timetable [33,34,38,39,41]. In contrast, conflicting priorities were found to be a barrier for some schools, where leadership teams were less actively involved and prioritised other tasks (often related to academic results) over the intervention [33,37,40,41].

Strong *leadership and communication* around the intervention were found to facilitate sustained delivery, with successful leaders making clear decisions regarding the interventions and communicating priorities to staff [33,34,36,40,41]. However, lack of communication and the resulting lack of awareness among staff about the programme could be a barrier to implementation, particularly with an intervention such as ‘First Step to Success’. This intervention involved teachers using a tool to identify students with high levels of anxiety and then referring them to school clinicians [36]. In this case, participants thought the lack of communication about what the intervention actually was and why it should be used may have led to insufficient numbers of referrals in the sustainability phase of their research [36].

#### 3.6.2. School Culture, Values, and Policies

Promotion of a *culture of support* in a school from the senior leadership team was also discussed as a facilitator to sustaining programmes, along with a general willingness to try new things [33,34,37]. For some, this meant the school leaders being involved in the programme and being supportive of the philosophy: “school administrators make the decisions about what classes to offer so teachers mentioned administrator’s support as crucial for ensuring the class could continue” [34] (p. 329). However, for others, support was more passive, with one school clinician saying that the senior staff were “very supportive of whatever I wanted to do. They didn’t particularly get involved or ask questions, they just let me run it again” [37] (p. 137). This leadership support also led to increased motivation and commitment from staff [39].

Another way for school leaders to demonstrate support for the programme was to make the *intervention part of school policy*, cementing commitment to the intervention [40,41]. In Dijkman et al.’s [41] study on the Good Behaviour Game (GBG), the GBG was mentioned in the policy plans of all the highly-sustained schools but in none of the weakly-sustained schools’ policy plans.

#### 3.6.3. Allocation of Resources

*Having a designated programme lead* was discussed both as a facilitator and a barrier to sustainability. A leadership-assigned programme coordinator or champion facilitated programme delivery by pushing for the programme to be implemented, promoting the programme, encouraging sustained use of the programme, and using relationships to overcome implementation barriers [33,40,41]. One of the participants in Dijkman et al.’s study highlighted this fundamental role as a coordinator for the Good Behaviour Game: “Honestly, I think that if it was not one of my tasks, the GBG would have just fallen over. No coordination – no GBG in the school” [41] (p. 86). However, the role of programme coordinator only worked if the individual staff member had enough allocated time to fulfil their responsibilities and stayed in the same role; champion staff turnover was identified as a barrier to continued delivery [33,41]. 

School leaders were also important for sustainability through their provision of *practical support*, such as scheduling the intervention into the timetable, providing rooms for intervention delivery (e.g., a private office or a space for group activities) and access to computers and technical resources [33,34,35,37,39]. Allocating *time for training* was also identified as a facilitator, with teachers needing to be released from other duties to attend training sessions and some schools planning annual training for staff [33,34,36,41].

### 3.7. Staff Engagement

Staff engagement in the delivery of interventions was the only factor discussed in every article in this review, with motivated staff contributing to the sustainability of interventions in some cases and a lack of engagement creating barriers for delivery in others. This factor is broken down into four themes below, with subthemes italicised in the text.

#### 3.7.1. Commitment from Individuals

Sustainability was also facilitated by *individual effort from staff members*, with some teachers and school staff making adaptations in order to continue intervention delivery [33,34,37,40,41]. In Crane et al. [33], mental health staff set up peer consultation to troubleshoot difficulties, and in Friend et al. [34] teachers did not have time in their week to deliver 1:1 counselling meetings for pupils, but instead incorporated individual meetings into the class. Some clinicians in Nadeem and Ringle’s [37] article also mentioned adapting the intervention resources slightly to engage the students more effectively (e.g., use of additional role plays or games). Similarly, in Dijkman et al.’s [41] study on GBG, the ability and willingness to take the initiative and make adaptations were found to be a key difference between the schools with weaker sustainability and those that sustained the GBG. Many schools perceived the intervention to be less suitable for children ≤6 years and ≥10 years, but in highly sustained schools the teachers worked with the GBG trainer to make adaptations, enabling the continuation of the intervention. In contrast, teachers in schools with weaker sustainability scores stopped using the programme completely when they encountered a problem [41]. With targeted interventions, it was also important for teachers to be involved in the process and take an active role in identifying and referring students [33,34,36]. 

School staff reported *enjoying delivery* of the interventions, stating that they were motivated to continue because the classes and sessions were fun to teach and in some cases made a pleasing change from normal lessons [34,37,38,39]. Additionally, individual staff members also contributed to successful continuation by *allowing time out of class* for pupils to receive the interventions, and in some cases even walked the child to their intervention session [33]. Conversely, a lack of willingness for pupils to miss lessons was found to be a barrier to sustainability, with some teachers “protective” over students’ time [33,37].

#### 3.7.2. Staff Turnover

Turnover of staff who were trained in intervention delivery was referenced as a key barrier to sustainability [33,34,36,37,39,41]. In one instance, a successfully sustaining school had provided training for new members of staff to introduce them to the principles of the programme [40]. However, in most cases the lack of availability to send new members of staff on training greatly reduced the capacity of a school to deliver these mental health interventions. For some schools, staff turnover contributed to lower intervention fidelity, as teachers who had not received training or materials were delivering only parts of the intervention [34], while in other instances, the programme was completely discontinued [33,36,37].

#### 3.7.3. Perceived Benefit for Pupils

Staff perceiving benefit for pupils was identified solely as a facilitator to sustainability. While some studies reported that school staff had seen the benefit of the intervention with regard to the *mental health and wellbeing* of the pupils [34,37,39], the most frequently reported benefit was pupil *behaviour and classroom climate* [34,35,37,39,41]. In Dijkman et al.’s [41] research, noticing results in terms of improved behaviour and a more positive classroom climate made teachers more willing to continue delivery. This study also referenced improved *academic performance* as an incentive for schools to continue delivering the intervention [41]. More *general benefits for pupils* were also referenced, with schools saying they had experienced a range of positive outcomes and benefits for their pupils, motivating staff to maintain intervention activities [35,37,38,42]. This was highlighted in LoCurto et al.’s [38] quantitative study, where regression analyses were used to explore predictors of sustained use. Along with scales measuring the acceptability and difficulty of delivering the intervention, a statistically significant predictor was that clinicians with more positive beliefs that the intervention improved their students’ outcomes were more likely to continue to use the treatment [38]. 

#### 3.7.4. Pupil and Parent Engagement

*Pupil engagement in the intervention* was a facilitator to sustainability as staff were more likely to continue delivery when they thought pupils were enjoying and engaging in the sessions [33,34,35,36,37,39]. In contrast, low levels of engagement were a barrier to sustainability. This was noted in Friend et al. [34], where low levels of pupil motivation were a barrier, and in Crane et al. [33], where some pupils’ behavioural problems or the severity of their anxiety symptoms made it too difficult for them to engage in the programme. 

Three studies referenced *parent participation* as an additional influence on sustainability; parent willingness to complete questionnaires and work with the school was a facilitator, but engaging parents for consent and treatment could also pose a challenge [33,36,37].

### 3.8. Intervention Characteristics

The different characteristics of each intervention were frequently cited in participants’ discussions around sustainability; the content, training opportunities, intervention materials and resources were all found to influence schools’ use of the intervention. This is broken down into three themes below, with additional subthemes italicised in the text.

#### 3.8.1. Content and Design

The design of interventions was referenced in multiple studies, with the *acceptability of interventions for staff* highlighted as a facilitator to sustainability. Programme materials that were more acceptable and less difficult to implement were more likely to result in continued use [33,37,38,39]. In LoCurto et al.’s [38] study, one of the strongest predictors was staff perceptions of the intervention materials: “clinicians who perceived the intervention materials as more acceptable, (i.e., easy to use, realistic/practical and fun to teach) and less difficult to implement, were more likely to report continued use” [38] (p. 686). Similarly, the *practicality and ease of use* of each intervention impacted sustainability. Interventions that were sustained were described as “easy to use/implement”, “manageable” and “well organised” [33,35,38]. Conversely, some elements of interventions were not deemed to be practical, such as the lengthy screening tool in CCAL [33] and the time required out of class for pupils receiving CBITS [37]; these both created challenges for sustainability. 

#### 3.8.2. Quality of Materials and Training

The quality of intervention materials was cited as a facilitator to sustainability, with school staff noting the importance of ready-made sessions and professionally prepared resources [33,37,39]. Similarly, having sufficient training in intervention delivery was found to be a facilitator for some with school staff feeling confident and ready for delivery [34,36,39], and others suggesting more training was required [33].

#### 3.8.3. Meeting Need and Fitting with School

The intervention meeting a need within a school influenced the likelihood that it would be sustained, with staff highlighting the ongoing need for the programme as a key facilitator [33,34,37,41]. This was demonstrated in Dijkman et al.’s [41] research, where schools with high sustainability explained that the programme was needed to continue to solve behavioural problems in the school, whereas schools that were not sustaining the GBG felt this need was no longer there. This is highlighted by one of the participants in Dijkman et al.’s [41] study, who explained: “The most important reason for not doing it anymore is that the necessity is gone. A lot of teachers left and new teachers came. These new ones are another type of teacher, they don′t need it anymore” [41] (p. 85).

### 3.9. Resources

A barrier to sustainability mentioned in nearly all included articles was access to resources, both in relation to staff capacity and funding. This is broken down into two main themes below, and the subthemes are italicised in the text.

#### 3.9.1. Staff Capacity

A frequent barrier to sustainability was staff having enough capacity to facilitate intervention delivery. *Competing priorities and responsibilities* for school staff often led to challenges, with some intervention activities going undelivered or being delivered with less consistency than during initial implementation [33,34,35,37,39,41]. Examples of this include the cessation of lunchtime activities due to other staff responsibilities [34], and intervention coordinators being unable to provide adequate supervision for teachers [41]. In two articles, school clinicians reported having less time for direct therapy as a result of competing priorities such as administrative tasks, psychological testing, and crisis management [33,37].

*Class sizes and caseloads* were also barriers to sustainability, with class sizes that were either too large or too small posing a challenge; large class sizes created difficulties with classroom management, but small groups were not sustainable as it was hard to justify offering the class [34]. Caseloads were also cited as a factor affecting sustainability; clinicians with smaller caseloads found it easier to continue the use of interventions, and higher caseloads were cited as one of the key reasons for stopping delivery [37,38]. 

#### 3.9.2. Funding

Lack of funding and appropriate resources were mentioned as barriers to delivering the interventions. If activities could be integrated easily into the school’s usual provision or the specific duties of a staff member, lack of funding for an intervention posed less of a problem [41]. However, activities that required additional funds, such as hiring guest instructors to deliver sessions or paying for staff and parents to receive training, were not sustained [34,36]. In the case of the total de-adoption of one intervention, a budget crisis at a higher level led to significant job restructuring and staff layoffs which resulted in the programme being cut [37]. 

### 3.10. External Support

While most of the included articles focused on sustainability at the school level, some higher-level factors were also discussed. The most salient factor was external support for interventions, which was found to be both a barrier in some instances and a facilitator in others. This factor is broken down into three themes below.

#### 3.10.1. District Support

Two articles from the U.S. discussed district-level support (similar to local authority level in England) as an important facilitator for sustainability. Loman et al. [36] observed that most of the schools that sustained implementation of First Steps to Success adopted the programme as a part of a districtwide initiative, while the non-sustaining schools initiated the programme independently. District infrastructure, coordination, and leadership all contributed to the likelihood of a school continuing delivery of an intervention [36,37]. 

#### 3.10.2. Consistency and Shifting Priorities

While support at the district level could facilitate sustainability, this was found to be dependent on specific personnel, and schools reported district-level leadership changes as a barrier to continuing delivery. A participant in Nadeem and Ringle’s [37] study explained that “without someone from the top supporting it and paving the way, it was very difficult to use [Cognitive Behavioural Intervention for Trauma in Schools] again” [37] (p. 138). With new leadership came shifting priorities; school clinicians reported that the focus had moved away from the programme, often onto academic success [37]. Similarly, Loman et al. [36] found that when key personnel moved from the district, the intervention quickly ceased to be implemented. 

#### 3.10.3. Higher-Level Support

One article mentioned that political endorsement of the prevention effort would be helpful, particularly when it came to school staff defending the intervention in front of colleagues [39].

## 4. Discussion

Given the increasing emphasis on schools to provide mental health education and support for children and young people, as well as significant local and national investment in this support, the aim of this research was to systematically review the factors affecting the sustainability of school-based mental health and emotional wellbeing programmes. The literature searches retrieved articles on both targeted and universal mental health interventions trialled in schools. These articles included a range of barriers and facilitators to sustained delivery. While some wider system-level factors were noted, most sustainability factors discussed in included articles were at school level, particularly in relation to school staff and leadership. Key facilitators to sustainability were leadership support and school staff members’ perceived benefit of the intervention for pupils, while key barriers included staff turnover, capacity, and competing priorities. 

Some themes were similar across both the school and wider system level—most notably, the importance of consistency and limited turnover of key personnel. Turnover amongst individual teachers (who had received intervention training), programme coordinators, the senior leadership team of a school, and even a district created a considerable barrier to sustainability. This is consistent with the broader literature on sustaining programmes in schools, where staffing issues are noted as one of the major barriers to sustainment [48,49,50]. Similarly, commitment and prioritisation across all levels of staff was a facilitator to sustained delivery of the mental health and wellbeing programmes in this review. This maps onto findings that identify continued engagement at all stakeholder levels as crucial [48,50,51]. For interventions to be sustained in schools, maintained commitment and prioritisation are required at all levels of the school system. 

In addition, several barriers and facilitators identified here for mental health and wellbeing interventions are consistent with previous reviews. For example, Herlitz et al.’s [19] review on public health interventions in schools and Askell-Williams and Koh’s [51] review on the sustainability of school initiatives both note the importance of school leadership support, staff turnover, perceived effectiveness, funding and resources, school policies and plans, belief in intervention, and fit with the school. This indicates that these themes may be central to sustainability irrespective of the nature of the intervention being implemented. However, there were some departures between the current review and those carried out previously. For instance, in both the Herlitz et al. [19] review and a recent qualitative study [48], a lack of confidence in school staff to deliver health promotion was a barrier to sustainability which did not feature in the current review. In contrast, while the importance of training was noted in the current review, there was a greater emphasis on staff enjoying delivering the sessions and simply having the capacity to do so. It is also perhaps surprising that the self-efficacy of staff which was highlighted in previous reviews did not come up in this review given the potentially sensitive content of these interventions. However, this may be due to schools’ growing remit to provide programmes around mental health and wellbeing, or perhaps it is the result of a wider cultural shift towards openness and discussion on these topics in Western societies [52,53]. 

The importance of evaluation and feedback around the intervention has also been cited as a key factor affecting sustainability in previous work [51], yet this was not observed here. In their development of a framework for sustainable implementation specifically relevant to educational contexts, Askell-Williams and Koh [51] highlight the need for such data collection but note that it often seems to be an afterthought. While the literature recommends the analysis of good quality implementation and sustainability data, the findings from the current review also show that this may be a gap as these data are not regularly collected by schools.

### 4.1. Strengths and Limitations

A strength of this review was that it used a broad definition of mental health and emotional wellbeing to capture a broad range of different types of school-based intervention, including both targeted and universal programmes. This allowed for a wide exploration of barriers and facilitators to sustainability in school settings pertaining to mental health and emotional wellbeing. This review included the rigorous double screening of titles, abstracts, and full texts (both with high kappa statistics) and the involvement of multiple researchers in thematic synthesis. This mitigates the risk of systematic bias at the screening stages whilst also decreasing the total number of errors or missed studies [54]. 

There are some limitations to this review. Despite attempts to conduct a comprehensive and broad search, with any systematic review there is a possibility that all relevant literature is not captured. This may be particularly relevant to sustainability as the construct is not well defined in the literature. To limit this, experts in the field were consulted and the reference sections of full-text articles were searched. However, it is possible that articles may have used a different synonym from those included in this search. In addition, the articles in this review have been limited to those published in English, excluding potentially relevant studies that may have been published in other languages. 

To explore the sustainability of these interventions in schools, stringent criteria were employed regarding research being conducted after the initial implementation period when external support and funding had finished. As a consequence, some papers on the sustainability of mental health programmes in schools were not included in this review as their models either explicitly involved continued external support for schools, or it was not possible to discern what schools in the sample had received in terms of district-level support or funding (e.g., Arnold et al., 2021; McIntosh et al., 2016; and Pinkelman et al., 2015). In the research conducted by Ertesvåg et al. [40], school staff were interviewed both toward the end of the initial programme period and again two and half years later. However, in the results section of this paper, the authors do not distinguish between the two data collection timepoints. Therefore, it is not possible to ascertain which themes are specifically relevant to the research question of this review. In order not to omit potentially relevant information for this review, Ertesvåg et al.’s [40] findings were included in the thematic analysis, and the barriers and facilitators identified were included in the discussion of the results, whilst also noted in grey at the end of Table 4. 

### 4.2. Recommendations

While not included in this review, models involving continued external support for schools may be an important pathway for sustained delivery of mental health interventions, and this should be explored further. The issue of staff turnover and shifting priorities at all levels of school systems is also an important area of focus, particularly as sustainment seems to be driven forward by individual members of school staff with little or no consideration of a wider network of responsibility and support. Crane et al. [33] set out to interview members of school staff at three timepoints but encountered low response rates at Year 3 due to staff turnover and participant attrition. In this instance, staff turnover proved to be a barrier to the research as well as to sustaining the intervention.

The articles in this review all pertain to schools in high-income countries where greater resources and capacity are available. With evidence of effective school-based mental health interventions also being delivered in low-income and middle-income countries, research into sustainability in different settings is crucial [55]. 

In keeping with Wiltsey-Stirman et al.’s [18] review on the sustainability of interventions, over half of the included studies did not include definitions of sustainability. Future studies should define sustainability and draw on implementation and sustainability frameworks to shape their research. For example, it would also be useful to underpin future research with a framework such as the Theoretical Domains Framework, which draws on behaviour change theories [56]. This framework approach could allow for further understanding of factors that may improve the implementation and sustainability of these interventions and assist with the development of practical solutions to some of the challenges.

In this review, we found that the perceived benefit of the interventions by school staff was a facilitator to sustainability, but it was not possible to draw conclusions about a link between effectiveness data regarding the interventions and sustained use. As in Herlitz et al.’s [19] review, there were gaps in reporting evidence of effectiveness and sustainability. This is a key question for future research and must be considered carefully when designing intervention trials.

Although a number of studies evaluated interventions at two timepoints (initial implementation and sustainability), only one of the research designs included here explored the process of sustainability over multiple timepoints [37]. This highlights a gap in understanding sustainability not only as an outcome (where activities are maintained) but also as a complex process involving adaptation and development in response to the emerging needs of a given system [57]. To explore sustainability as a process, future research would benefit from stronger designs and methodology, particularly longitudinal research involving multiple timepoints.

## 5. Conclusions

There is little high-quality research on the sustainability of mental health and emotional wellbeing interventions in schools. Although there are promising findings on the effectiveness of some school-based interventions for mental health [9], research on long-term implementation and sustainability is very limited. Despite this, a range of barriers and facilitators to sustaining these types of intervention in schools have been identified in this review. The majority of these barriers and facilitators to mental health interventions are at the school level and are very similar to health interventions and educational improvement interventions more generally [19,51]. Given this, it is important for sustainability researchers to focus broadly on the difficulties of delivering interventions in school settings and the unique challenges of working within such complex systems [58,59]. Barriers such as constantly shifting priorities and high levels of staff turnover may be particularly salient in schools and consequently would require specific approaches to increase sustainability. 

In the U.K., there has been a significant policy shift communicating that pupils’ mental health and emotional wellbeing falls at least partly in schools’ remit. This has paved the way for greater infrastructure to provide mental health support in schools. It is yet to be established whether these changes might remove some of the barriers described in this review and improve the capacity of schools to sustain these types of interventions. While interventions becoming part of school culture and values may facilitate sustainability, the ever-pressing concerns around logistics, limited time, and capacity in schools suggest that more is required from leaders and policymakers for mental health and wellbeing interventions to be successfully sustained. 

## Figures and Tables

**Table 1:** Interventions included in the review.

Article No.	Author, Year of Publication, Country	Intervention Name	Intervention Aim(s)	Pupil Age, Type of Intervention	Intervention Description/Components	Intervention Deliverer(s)	Intervention Training and Supervision	Intervention Resources	Reported Evidence of Effectiveness (Before Sustainability Evaluation)
1	Adametz, 2017, Germany	PriMa	To reduce risk factors of anorexia	11–13 years old, targeted (girls only)	9 sessions (45–90 min) including role plays, analyses of film sequences, poster discussions	School teachers or school social workers	1-day training session	100-page teaching manual with workbooks for pupils	Positive impact on body self-esteem, life skills, healthy eating behaviour, and classroom climate
		Torera	To reduce risk factors of bulimia and binge eating disorder	12–14 years old, universal	Focus on protective factors, e.g., self-esteem				
2	Crane, 2021, U.S.	Camp Cope-A-Lot (CCAL)	To treat youth with generalised anxiety disorder, social anxiety disorder and separation anxiety disorder	7–13 years old, targeted	12 sessions including computer-assisted relaxation training, cognitive restructuring and problem solving, followed by tailored exposure tasks	School staff	1-day training workshop, weekly group consultation calls for first 3 months	Coach’s manual, workbooks for pupils	*Not reported for these schools, but has been found to demonstrate efficacy in anxiety symptom reduction* [43]
3	Dijkman, 2017, The Netherlands	Good Behaviour Game (GBG)	To reinforce pro-social behaviour and reduce aggressive and disruptive behaviour	Primary schools (5–11 years old), universal	Three times a week for 15 min at start of the year, time increased gradually throughout the academic year	School teachers	Three ½-day training sessions, coaching (10 classroom observations with feedback)	Pictograms and cards used in classrooms	*Not reported for these schools, but shown to be effective in preventing and reducing behavioural problems in the classroom, and has positive long-term effects on smoking, drug and alcohol abuse, antisocial personality disorder, and violent and criminal behaviour* [44,45,46,47]
4	Ertesvåg, 2010, Norway	Respect Program	To reduce problem behaviour, particularly disobedience, off-task behaviour and bullying	11–16 years old, universal	Whole school approach, project group	School staff	2-day seminar for project group (management and key personnel), 1-day workshop for all staff, mentoring (4–6 meetings per year), monthly peer-counselling sessions	*No detail provided*	Decrease in problem behaviours, small to moderate effect sizes for most grade levels
5	Friend, 2014, U.S.	New Moves	To address the needs of adolescent girls at risk for weight-related problems	High school (14–18 years old), targeted (girls only)	All-girl physical education classes 4 days a week, classroom sessions on nutrition and social support, individual counselling sessions, lunch get-togethers	School PE teachers, community guest instructors, New Moves intervention staff (classroom sessions and 1:1 counselling)	1-day training at the start, ½-day training in the middle of the program, ongoing support from New Moves staff	Teacher guidebook and curriculum, workbook for pupils, recipe cards, community resources, postcards to send to parents	Improvements were seen for sedentary activity, eating patterns, unhealthy weight control behaviours, and body/self-image
6	Jolivette, 2014, U.S.	School-Wide Positive Behavioural Interventions and Supports (SWPBIS)	To address problem behaviour	7–17 years old, universal (residential school)	Preventative three-tiered behavioural framework (whole-school expectations, classroom and small group interventions, individualised support)	School staff	1-day planning training, school administrator training in producing SWIS reports	School-wide information systems (SWIS) to monitor behaviour	Decreased number of discipline referrals and decreased number of students accruing referrals
7	LoCurto, 2020, U.S.	Modular CBT (M-CBT)	To reduce anxiety symptoms and severity	6–18 years old, targeted	12 individual sessions, seven core modules incl. psychoeducation, problem solving, exposure, relaxation skills	School-based clinicians	1-day training in M-CBT, training to use the SCARED screening questionnaire, assigned clinical supervisor	Treatment manual, forms, handouts, case summary	No significant treatment main effects on primary outcomes, parent-report of child anxiety showed greater improvements in CBT relative to treatment as usual
8	Loman, 2010, U.S.	First Step to Success (FSS)	To divert problem behaviour patterns	Primary school (5–8 years old), targeted	Screening procedure, behavioural intervention with teacher, child, parents and peers	School coach (ideally psychologist/ counsellor) and teachers	1 and 2-day training sessions	*No detail provided*	Significant pre–post behavioural changes in adaptive, aggression, maladaptive, and academic engaged time measures
9	Nadeem, 2017, U.S.	Cognitive Behavioural Intervention for Trauma in Schools (CBITS)	To reduce psychological symptoms related to traumatic stress, anxiety, and depression	11 years old, targeted	Brief screening tool, 10-session group intervention, 1–3 individual sessions, core CBT techniques including psychoeducation, relaxation, exposure, problem solving	School clinicians	Formal training, implementation support groups	Implementation manual, report provided at end of the year	Significant pre–post intervention decline in PTSD symptoms
10	Ruby, 2019, U.K.	The Boxall Profile	To improve school support for social, emotional, and mental health needs	Primary school (5–11 years old), universal	Psychosocial assessment tool to accurately determine pupils’ social and emotional functioning and wellbeing	Teachers/school staff	2-day training, termly network support meetings	Online Boxall Profile tool, automatically generated data	Approach was found to be feasible, valuable, and effective at identifying and triggering support for children with SEMH needs

**Table 2:** Conceptualising sustainability.

Article Number	Lead Author, Year of Publication, Country	Sustainability Term Used	Sustainability Definition	Implementation or Sustainability Framework Discussed	Time between Initial Implementation Period and Sustainability Evaluation
1	Adametz, 2017, Germany	Long-term implementation	*No definition provided*	*No framework referenced*	>8 years
2	Crane, 2021, U.S.	Sustainability	*No definition provided*	Consolidated Framework for Implementation Research (CFIR; Damschroder et al., 2009)	1 year
3	Dijkman, 2017, The Netherlands	Sustainability	“sustainability means that the program is incorporated into the organisation and has become a stable and regular part of organisational procedures and behaviour” p. 81	Theoretical framework based on Pluye et al. (2004)	2 years
4	Ertesvåg, 2010, Norway	Continuation	“The term ‘continuation’ refers to the work after the program period when external project support has ceased and the schools are supposed to continue the work on their own’’ p. 326	Educational change (Fullan, 2007)	2.5 years
5	Friend, 2014, U.S.	Sustainability	*No definition provided*	*No framework referenced*	1–2 years
6	Jolivette, 2014, U.S.	Maintenance	*No definition provided*	*No framework referenced*	6 months
7	LoCurto, 2020, U.S.	Sustained use	*No definition provided*	Diffusion of innovations theory (DOI; Rogers, 2003); exploration, preparation, implementation and sustainment (EPIS; Aarons, Hurlburt, and Horwitz, 2011)	3.4 years
8	Loman, 2010, U.S.	Sustainability	“the continued implementation of a practice at a level of fidelity that continues to produce intended benefits” p. 179	Logic model for sustainability presented by McIntosh et al. (2009)	Up to 10 years
9	Nadeem, 2017, U.S.	De-adoption	“Sustainment can be defined as the maintenance of EBPs ‘for the continued achievement of desirable program and population outcomes’ (Scheirer and Dearing, 2011; p. 2060). De-adoption, on the other hand, can occur at any stage of the implementation process, and often refers to failure to sustain an EBP.” p. 2	Conceptual framework for sustainability (Scheirer and Dearing, 2011); conceptual model of evidence-based implementation (Aarons, Hurlburt, and Horwitz, 2011); implementation framework (Domitrovich et al., 2008); implementation framework (Fixsen et al., 2013)	2 years
10	Ruby, 2019, U.K.	Sustainability	*No definition provided*	*No framework referenced*	8 months

**Table 3:** Study design and quality assessment.

Article Number	Lead Author, Year of Publication, Country	Study Design	Study Participants	Quality Assessment Score
**1**	Adametz, 2017, Germany	Qualitative—interviews	Teachers involved in intervention delivery, headteachers and a social worker	High
**2**	Crane, 2021, U.S.	Qualitative—interviews	School staff	High
**3**	Dijkman, 2017, The Netherlands	Mixed methods—interviews and 20-item checklist	School staff—GBG coordinators	Medium
**4**	Ertesvåg, 2010, Norway	Qualitative—interviews	School staff—project groups	Medium
**5**	Friend, 2014, U.S.	Mixed methods—interviews, survey, and PE lesson observation	Teachers involved in intervention delivery	Medium
**6**	Jolivette, 2014, U.S.	Mixed methods case study—process monitoring data and focus group	School staff	Low
**7**	LoCurto, 2020, U.S.	Quantitative—survey	School clinicians	Medium
**8**	Loman, 2010, U.S.	Quantitative—survey	School staff (including headteachers, classroom teachers, and school psychologists)	Medium
**9**	Nadeem, 2017, U.S.	Qualitative—interviews	School clinicians	High
**10**	Ruby, 2019, U.K.	Qualitative—interviews	*Not provided*	Low

**Table 4:** Barriers and facilitators to sustaining mental health programmes in schools.

Sustainability Level	Factors	Themes	Subthemes	Adametz et al. (2017)	Crane et al. (2021)	Dijkman et al. (2017)	Friend et al. (2014)	Jolivette et al. (2014)	LoCurto et al. (2020)	Loman et al. (2010)	Nadeem and Ringle (2016)	Ruby et al. (2019)	Ertesvåg et al. (2010) †
***School Level***	**1. School leadership**	**1.1 Support and prioritisation**	Prioritising the intervention	**+**	**−**	**+/−**	**+**		**+**		**−**		**+/−**
			Leadership and communication		**−**	**+/−**				**+**	**+/−**		**+/−**
		**1.2 School culture, values, and policies**	Culture of support	**+**	**+**		**+**				**+**		**+/−**
			Intervention part of school policy			**+/−**							**+/−**
		**1.3 Allocation of resources**	Having a designated programme lead		**+/−**	**+/−**							
			Practical support	**+**	**+**		**+**	**+**		**+**	**+**		**+/−**
			Time for training		**+**	**−**	**−**			**+**			**+**
	**2. Staff engagement**	**2.1 Commitment from individuals**	Individual effort from staff members	**−**	**+**	**+**	**+**	**−**		**+/−**	**+**		**−**
			Staff enjoying delivery	**+**	**+/−**		**+**		**+**		**+**		
			Staff allowing time out of class		**+/−**						**−**		
		**2.2 Staff turnover**	**-**	**−**	**−**	**−**	**−**			**−**	**−**		**−**
		**2.3 Perceived benefit for pupils**	Academic performance			**+**							
			Behaviour and classroom climate	**+**		**+**	**+**	**+**			**+**		
			Mental health and wellbeing	**+**			**+**				**+**		
			General benefits for pupils					**+**	**+**		**+**	**+**	
		**2.4 Pupil and parent engagement**	Pupil engagement in the intervention	**+**	**+/−**		**−**	**+/−**	**+**		**+**		
			Parent participation		**+**					**+**	**−**		**+**
	**3. Intervention characteristics**	**3.1 Content and design**	Acceptability of intervention for staff	**+**	**+/−**				**+**		**+**		
			Practicality and ease of use	**+**	**+/−**			**+**	**+**		**+/−**		
		**3.2 Quality of materials and training**	**-**	**+**	**+/−**		**+**						
		**3.3 Meeting need and fitting with school**	**-**		**+**	**+/−**	**+**				**+**		
	**4. Resources**	**4.1 Capacity**	Competing priorities and responsibilities	**−**	**−**	**−**	**−**	**−**	**−**		**−**		
			Class size and caseloads				**−**		**+/−**		**+/−**		
		**4.2 Funding**	**-**			**+**				**−**	**−**		
***Wider System Level***	**5. External support**	**5.1 District support**	**-**							**+/−**	**+/−**		
		**5.2 Consistency and shifting priorities**	**-**							**−**	**−**		
		**5.3 Higher level support**	**-**	**+**									

+ = facilitator, − = barrier, +/− = discussed as both a barrier and a facilitator. † Ertesvåg et al. [40] do not distinguish between two data collection timepoints (initial implementation and sustainability follow-up), and consequently, it is not possible to isolate factors specific to sustainability (see Section 4.1 for details).

## Data Availability

Not applicable.

## Supplementary Materials

The following supporting information can be downloaded at: https://www.mdpi.com/article/10.3390/ijerph19063587/s1, File S1 Example search strategy and key websites searched.
