# Supplementary material for: Barriers and Facilitators to Sustaining School-Based Mental Health and Wellbeing Interventions: A Systematic Review
Source: Int J Environ Res Public Health. 2022 Mar 17;19(6):3587. doi: 10.3390/ijerph19063587 (PMC8949982; doi:10.3390/ijerph19063587)
Supplement: Supplementary file 1 [file ijerph-19-03587-s001.zip › ijerph-1627905-supplementary.pdf]

File S1 – Example Search Strategy

Database: APA PsycInfo <1806 to February Week 4 2021>

Search Strategy:

----

1 (sustain* or continua* or maintenance or institutionali?ation or routini?ation or embed* or incorporation or integration or normali?ation or stabili?ation or durab* or long-term implementation or (long term adj4 implementation) or discontinua* or scale-up or scaling-up or endurance or persistence or de-adopt*).ab,ti. (331169)

2 school based intervention/ (19397)

3 (intervention* or program* or organi?ational change* or organi?ational transformation* or change process* or innovation* or initiative*).ab,ti. (769779)

4 exp program evaluation/ (20782)

5 2 or 3 or 4 (775791)

6 (school* or pupil* or teacher* or school-based).ab,ti. (494406)

7 schools/ or boarding schools/ or charter schools/ or colleges/ or elementary schools/ or high schools/ or institutional schools/ or junior high schools/ or middle schools/ or military schools/ or nongraded schools/ or technical schools/ (64608)

8 students/ or exp elementary school students/ or high school students/ or junior high school students/ or kindergarten students/ or middle school students/ or special education students/ (122787)

9 teachers/ or elementary school teachers/ or high school teachers/ or junior high school teachers/ or middle school teachers/ or special education teachers/ or exp educational personnel/ (122722)

10 6 or 7 or 8 or 9 (564437)

11 mental disorders/ or exp affective disorders/ or exp anxiety disorders/ or exp bipolar disorder/ or borderline states/ or exp chronic mental illness/ or exp dissociative disorders/ or exp eating disorders/ or gender dysphoria/ or mental disorders due to general medical conditions/ or exp neurosis/ or exp paraphilias/ or exp personality disorders/ or exp psychosis/ or serious mental illness/ or exp sleep wake disorders/ or exp somatoform disorders/ or exp "stress and trauma related disorders"/ or exp "substance related and addictive disorders"/ or exp thought disturbances/ or exp emotional adjustment/ or exp well being/ (716631)

12 (mental health or mental disorder* or mental* fit* or mental illness* or wellbeing or well being or emotional difficult* or emotional problem* or emotional adjustment or emotional regulation or emotional disorder* or affective disorder* or anxiety or anxious* or psychos* or neuros* or depress* or panic* or phobia* or self harm or self-harm or bipolar or

eating disorder* or eating difficult* or anorex* or bulimi* or EDNOS or trauma* or PTSD or post traumatic stress disorder* or suicid* or delusion* or behav* dis* or behav* problem* or behav* issue* or behav* difficult* or peer difficult* or peer problem* or relationship difficult* or relationship problem* or relationship issue* or family problem* or family issue* or family difficult* or conduct disorder* or oppositional defiant disorder* or antisocial personality disorder* or aggressi* or inattention or hyperactivity or substance abuse* or substance misuse* or drug abuse* or drug misuse* or alcohol abuse* or alcohol misuse* or internalis* or internaliz* or externalis* or externaliz*).ab,ti. (1167985)

13 11 or 12 (1392695)

14 1 and 5 and 10 and 13 (3047)

15 limit 14 to (english language and yr="2000 -Current") (2446)

****

Key websites searched

Australian Health Promoting Schools Association

Barnardo's

Communities and Schools Promoting Health

Education Endowment Foundation

EPPI-Centre database of education research

Evidence Based Practice Unit

Institute for Effective Education

International School Health Network

International Union for Health Promotion and Education

Mental Health Foundation

National Centre for Social Research

National Foundation for Education Research

Nurture UK

Place2Be

School Health Education Unit

Schools for Health in Europe website

WHO
